# Supplementary material for: Personalized single-cell networks: a framework to predict the response of any gene to any drug for any patient
Source: BioData Min. 2021 Aug 5;14:37. doi: 10.1186/s13040-021-00263-w (PMC8340371; doi:10.1186/s13040-021-00263-w)
Supplement: Supplementary file 1 — Additional file 1 Appendix. [file 13040_2021_263_MOESM1_ESM.zip › AppendixR1.pdf]

Appendix to:
Personalized single-cell networks: a framework to predict the response of any gene to any drug for any patient

Haripriya Harikumar[1,2+*], Thomas P. Quinn[1+*], Santu Rana[1], Sunil Gupta[1], and Svetha Venkatesh[1]

[1]Applied Artificial Intelligence Institute, Deakin University, Geelong, Australia
[2]Institute for Health Transformation, Deakin University, Geelong, Australia
+ contributed equally, * *h.harikumar@deakin.edu.au; contacttomquinn@gmail.com*

1 Overview

In an effort to bring greater clarity to the specific details of the methods, we have prepared an Appendix that includes a long-form analysis of toy data. The Appendix describes the example input data, intermediate transition probability matrix calculations, convergence plots, and step-wise recommendation scores. The toy data have analogy to the bipartite graphs and gene co-expression networks used in this paper, but are smaller in dimension and therefore serve as a better illustration of the method.

2 Examining the input data

Let us consider a toy example. Table 1 shows a user-item matrix, where the value of each cell represents how much the user likes the item or not (adapted from [1]). The range of the response values are between [-5, 5], where -5 indicates the user highly dislikes the item and 5 indicates the user highly likes the item. In this example, user u_1 is similar to u_2 because they both have like the items i_1 and i_2. User u_1 is also similar to u_3 because they both dislike same items i_4 and i_5. This table defines a bipartite graph, connecting users to items. This bipartite graph is analogous to the gene-drug and gene-annotation bipartite graphs used in the paper. (Although not shown here, it is possible to extend Table 1 with information about the relationship between users. This would form a user-user graph that is analogous to the gene-gene graphs used in the paper.)

If we consider items i_5 and i_6, we could say that i_6 is the better choice for u_1 because u_1 and u_3 dislike the same items, and u_3 likes i_6. However, we could also say that i_5 is a bad choice for u_1 because u_1 and u_2 like the same items, and u_2 dislikes i_5. Single-channel RWR may make use of a weight-shifted graph – having only positive values in it. Here, u_1 and u_2 have more in common compared to u_3, and therefore i_5 may be recommended over i_6. This can be prevented with a dual channel network, where negative and positive signed values signify different kinds of importance.

	i1	i2	i3	i4	i5	i6
u1	1	2	-1	-2	0	0
u2	5	4	0	0	-1	0
u3	0	0	-3	-3	0	1

Table 1: Input data. Each row represents users, and each column represents items. The value of each cell represents how much the user likes the item or not. Data adapted from [1].

3 Computing the transition probability matrix

To perform RWR, we must compute the *transition probability matrix* of each user with respect to each item. Once computed, the direction of the walk is chosen based on the values in the transition probability matrix (i.e., the larger the value, the more likely it will be chosen for the random walk). Since we have two channels – one for positive and one for negative – we also have two transition probability matrices. These are shown in Tables 2 and 3. At step 0, the initial transition probability matrices reflect the positive and negative edge weights, respectively.

	i1	i2	i3	i4	i5	i6
u1	0.166667	0.333333	0	0	0	0
u2	0.5	0.4	0	0	0	0
u3	0	0	0	0	0	0.142857

Table 2: Transition probability matrix based on positive edge weights.

	i1	i2	i3	i4	i5	i6
u1	0	0	0.166667	0.333333	0	0
u2	0	0	0	0	0.1	0
u3	0	0	0.428571	0.428571	0	0

Table 3: Transition probability matrix based on negative edge weights.

4 Updating the transition probability matrix

Once the transition probability matrices are initialized, they are then used to compute the user-item *rank* (i.e., the relative preference a user might have for an item). This rank is computed for each user-item pair according to the formulas presented in the Methods of the paper. The ranks get re-computed iteratively at each step, for a pre-specified number of steps. In later steps, missing "zero" links will get filled with non-zero values. After the last step, the final ranks can be used to recommend new items to the users.

Tables 4, 5, and 6 show the updated ranks for each RWR iteration for users u_1, u_2, and u_3 (respectively). For this example, we set the number of steps to 50 and α to 0.1. Although the items i_5 and i_6 have 0 values for u_1 in the beginning, they get updated to non-zero values after the first step (by leveraging shared information according to the "guilt-by-association' assumption). The convergence plots for the u_1, u_2 and u_3 are shown in Figure 1. When the difference between the t-th updated probability to the $(t-1)$-th probability is 0, we say that the algorithm has converged.

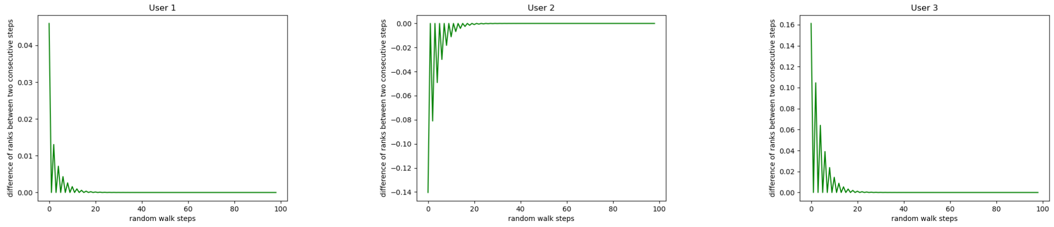

(a) User 1 (b) User 2 (c) User 3

Figure 1: Convergence plot of users u_1, u_2, and u_3 over 100 random walk iterations.

1	0.1667	0.3333	-0.1667	-0.3333	0.0	0.0
2	0.2203	0.2651	-0.1869	-0.2609	-0.0292	0.0376
3	0.2203	0.2651	-0.1869	-0.2609	-0.0292	0.0376
4	0.2267	0.2641	-0.185	-0.2539	-0.0316	0.0387
5	0.2267	0.2641	-0.185	-0.2539	-0.0316	0.0387
6	0.2292	0.2657	-0.1831	-0.2517	-0.0321	0.0382
7	0.2292	0.2657	-0.1831	-0.2517	-0.0321	0.0382
8	0.2307	0.2668	-0.1819	-0.2504	-0.0324	0.0378
9	0.2307	0.2668	-0.1819	-0.2504	-0.0324	0.0378
10	0.2315	0.2675	-0.1812	-0.2497	-0.0326	0.0375
11	0.2315	0.2675	-0.1812	-0.2497	-0.0326	0.0375
12	0.2321	0.2679	-0.1807	-0.2492	-0.0327	0.0374
13	0.2321	0.2679	-0.1807	-0.2492	-0.0327	0.0374
14	0.2324	0.2681	-0.1804	-0.2489	-0.0328	0.0373
15	0.2324	0.2681	-0.1804	-0.2489	-0.0328	0.0373
16	0.2326	0.2683	-0.1803	-0.2488	-0.0328	0.0373
17	0.2326	0.2683	-0.1803	-0.2488	-0.0328	0.0373
18	0.2327	0.2684	-0.1802	-0.2487	-0.0328	0.0372
19	0.2327	0.2684	-0.1802	-0.2487	-0.0328	0.0372
20	0.2328	0.2684	-0.1801	-0.2486	-0.0329	0.0372
21	0.2328	0.2684	-0.1801	-0.2486	-0.0329	0.0372
22	0.2328	0.2685	-0.1801	-0.2486	-0.0329	0.0372
23	0.2328	0.2685	-0.1801	-0.2486	-0.0329	0.0372
24	0.2329	0.2685	-0.1801	-0.2485	-0.0329	0.0372
25	0.2329	0.2685	-0.1801	-0.2485	-0.0329	0.0372
26	0.2329	0.2685	-0.18	-0.2485	-0.0329	0.0372
27	0.2329	0.2685	-0.18	-0.2485	-0.0329	0.0372
28	0.2329	0.2685	-0.18	-0.2485	-0.0329	0.0372
29	0.2329	0.2685	-0.18	-0.2485	-0.0329	0.0372
30	0.2329	0.2685	-0.18	-0.2485	-0.0329	0.0372
31	0.2329	0.2685	-0.18	-0.2485	-0.0329	0.0372
32	0.2329	0.2685	-0.18	-0.2485	-0.0329	0.0372
33	0.2329	0.2685	-0.18	-0.2485	-0.0329	0.0372
34	0.2329	0.2685	-0.18	-0.2485	-0.0329	0.0372
35	0.2329	0.2685	-0.18	-0.2485	-0.0329	0.0372
36	0.2329	0.2685	-0.18	-0.2485	-0.0329	0.0372
37	0.2329	0.2685	-0.18	-0.2485	-0.0329	0.0372
38	0.2329	0.2685	-0.18	-0.2485	-0.0329	0.0372
39	0.2329	0.2685	-0.18	-0.2485	-0.0329	0.0372
40	0.2329	0.2685	-0.18	-0.2485	-0.0329	0.0372
41	0.2329	0.2685	-0.18	-0.2485	-0.0329	0.0372
42	0.2329	0.2685	-0.18	-0.2485	-0.0329	0.0372
43	0.2329	0.2685	-0.18	-0.2485	-0.0329	0.0372
44	0.2329	0.2685	-0.18	-0.2485	-0.0329	0.0372
45	0.2329	0.2685	-0.18	-0.2485	-0.0329	0.0372
46	0.2329	0.2685	-0.18	-0.2485	-0.0329	0.0372
47	0.2329	0.2685	-0.18	-0.2485	-0.0329	0.0372
48	0.2329	0.2685	-0.18	-0.2485	-0.0329	0.0372
49	0.2329	0.2685	-0.18	-0.2485	-0.0329	0.0372
50	0.2329	0.2685	-0.18	-0.2485	-0.0329	0.0372

Table 4: The rank of items recommended for u_1 at each step.

1	0.5	0.4	0.0	0.0	-0.1	0.0
2	0.4415	0.3883	-0.0292	-0.0585	-0.0824	0.0
3	0.4415	0.3883	-0.0292	-0.0585	-0.0824	0.0
4	0.4138	0.3689	-0.0514	-0.0829	-0.0764	0.0066
5	0.4138	0.3689	-0.0514	-0.0829	-0.0764	0.0066
6	0.3973	0.3564	-0.0651	-0.0972	-0.073	0.011
7	0.3973	0.3564	-0.0651	-0.0972	-0.073	0.011
8	0.3873	0.3488	-0.0734	-0.1058	-0.071	0.0137
9	0.3873	0.3488	-0.0734	-0.1058	-0.071	0.0137
10	0.3812	0.3441	-0.0785	-0.1111	-0.0697	0.0153
11	0.3812	0.3441	-0.0785	-0.1111	-0.0697	0.0153
12	0.3775	0.3413	-0.0816	-0.1143	-0.069	0.0163
13	0.3775	0.3413	-0.0816	-0.1143	-0.069	0.0163
14	0.3753	0.3396	-0.0835	-0.1163	-0.0685	0.0169
15	0.3753	0.3396	-0.0835	-0.1163	-0.0685	0.0169
16	0.3739	0.3385	-0.0846	-0.1175	-0.0682	0.0173
17	0.3739	0.3385	-0.0846	-0.1175	-0.0682	0.0173
18	0.3731	0.3379	-0.0853	-0.1182	-0.068	0.0175
19	0.3731	0.3379	-0.0853	-0.1182	-0.068	0.0175
20	0.3725	0.3375	-0.0858	-0.1186	-0.0679	0.0176
21	0.3725	0.3375	-0.0858	-0.1186	-0.0679	0.0176
22	0.3722	0.3372	-0.086	-0.1189	-0.0679	0.0177
23	0.3722	0.3372	-0.086	-0.1189	-0.0679	0.0177
24	0.372	0.3371	-0.0862	-0.1191	-0.0678	0.0178
25	0.372	0.3371	-0.0862	-0.1191	-0.0678	0.0178
26	0.3719	0.337	-0.0863	-0.1192	-0.0678	0.0178
27	0.3719	0.337	-0.0863	-0.1192	-0.0678	0.0178
28	0.3719	0.3369	-0.0863	-0.1192	-0.0678	0.0178
29	0.3719	0.3369	-0.0863	-0.1192	-0.0678	0.0178
30	0.3718	0.3369	-0.0864	-0.1193	-0.0678	0.0178
31	0.3718	0.3369	-0.0864	-0.1193	-0.0678	0.0178
32	0.3718	0.3369	-0.0864	-0.1193	-0.0678	0.0178
33	0.3718	0.3369	-0.0864	-0.1193	-0.0678	0.0178
34	0.3718	0.3369	-0.0864	-0.1193	-0.0678	0.0178
35	0.3718	0.3369	-0.0864	-0.1193	-0.0678	0.0178
36	0.3718	0.3369	-0.0864	-0.1193	-0.0678	0.0178
37	0.3718	0.3369	-0.0864	-0.1193	-0.0678	0.0178
38	0.3718	0.3369	-0.0864	-0.1193	-0.0678	0.0178
39	0.3718	0.3369	-0.0864	-0.1193	-0.0678	0.0178
40	0.3718	0.3369	-0.0864	-0.1193	-0.0678	0.0179
41	0.3718	0.3369	-0.0864	-0.1193	-0.0678	0.0179
42	0.3718	0.3369	-0.0864	-0.1193	-0.0678	0.0179
43	0.3718	0.3369	-0.0864	-0.1193	-0.0678	0.0179
44	0.3718	0.3369	-0.0864	-0.1193	-0.0678	0.0179
45	0.3718	0.3369	-0.0864	-0.1193	-0.0678	0.0179
46	0.3718	0.3369	-0.0864	-0.1193	-0.0678	0.0179
47	0.3718	0.3369	-0.0864	-0.1193	-0.0678	0.0179
48	0.3718	0.3369	-0.0864	-0.1193	-0.0678	0.0179
49	0.3718	0.3369	-0.0864	-0.1193	-0.0678	0.0179
50	0.3718	0.3369	-0.0864	-0.1193	-0.0678	0.0179

Table 5: The rank of items recommended for u_2 at each step.

1	0.0	0.0	-0.4286	-0.4286	0.0	0.1429
2	0.0376	0.0752	-0.3695	-0.4071	0.0	0.1106
3	0.0376	0.0752	-0.3695	-0.4071	0.0	0.1106
4	0.0717	0.1038	-0.3395	-0.3782	-0.0066	0.1003
5	0.0717	0.1038	-0.3395	-0.3782	-0.0066	0.1003
6	0.093	0.1202	-0.3216	-0.3597	-0.011	0.0945
7	0.093	0.1202	-0.3216	-0.3597	-0.011	0.0945
8	0.1061	0.1302	-0.3106	-0.3484	-0.0137	0.091
9	0.1061	0.1302	-0.3106	-0.3484	-0.0137	0.091
10	0.114	0.1363	-0.304	-0.3415	-0.0153	0.0888
11	0.114	0.1363	-0.304	-0.3415	-0.0153	0.0888
12	0.1189	0.14	-0.3	-0.3374	-0.0163	0.0875
13	0.1189	0.14	-0.3	-0.3374	-0.0163	0.0875
14	0.1218	0.1422	-0.2975	-0.3348	-0.0169	0.0867
15	0.1218	0.1422	-0.2975	-0.3348	-0.0169	0.0867
16	0.1236	0.1436	-0.296	-0.3332	-0.0173	0.0862
17	0.1236	0.1436	-0.296	-0.3332	-0.0173	0.0862
18	0.1247	0.1445	-0.2951	-0.3323	-0.0175	0.0859
19	0.1247	0.1445	-0.2951	-0.3323	-0.0175	0.0859
20	0.1254	0.145	-0.2945	-0.3317	-0.0176	0.0858
21	0.1254	0.145	-0.2945	-0.3317	-0.0176	0.0858
22	0.1258	0.1453	-0.2942	-0.3314	-0.0177	0.0857
23	0.1258	0.1453	-0.2942	-0.3314	-0.0177	0.0857
24	0.1261	0.1455	-0.294	-0.3312	-0.0178	0.0856
25	0.1261	0.1455	-0.294	-0.3312	-0.0178	0.0856
26	0.1262	0.1456	-0.2938	-0.331	-0.0178	0.0856
27	0.1262	0.1456	-0.2938	-0.331	-0.0178	0.0856
28	0.1263	0.1457	-0.2938	-0.3309	-0.0178	0.0855
29	0.1263	0.1457	-0.2938	-0.3309	-0.0178	0.0855
30	0.1264	0.1457	-0.2937	-0.3309	-0.0178	0.0855
31	0.1264	0.1457	-0.2937	-0.3309	-0.0178	0.0855
32	0.1264	0.1457	-0.2937	-0.3309	-0.0178	0.0855
33	0.1264	0.1457	-0.2937	-0.3309	-0.0178	0.0855
34	0.1264	0.1457	-0.2937	-0.3308	-0.0178	0.0855
35	0.1264	0.1457	-0.2937	-0.3308	-0.0178	0.0855
36	0.1264	0.1458	-0.2937	-0.3308	-0.0178	0.0855
37	0.1264	0.1458	-0.2937	-0.3308	-0.0178	0.0855
38	0.1264	0.1458	-0.2936	-0.3308	-0.0178	0.0855
39	0.1264	0.1458	-0.2936	-0.3308	-0.0178	0.0855
40	0.1264	0.1458	-0.2936	-0.3308	-0.0179	0.0855
41	0.1264	0.1458	-0.2936	-0.3308	-0.0179	0.0855
42	0.1264	0.1458	-0.2936	-0.3308	-0.0179	0.0855
43	0.1264	0.1458	-0.2936	-0.3308	-0.0179	0.0855
44	0.1264	0.1458	-0.2936	-0.3308	-0.0179	0.0855
45	0.1264	0.1458	-0.2936	-0.3308	-0.0179	0.0855
46	0.1264	0.1458	-0.2936	-0.3308	-0.0179	0.0855
47	0.1264	0.1458	-0.2936	-0.3308	-0.0179	0.0855
48	0.1264	0.1458	-0.2936	-0.3308	-0.0179	0.0855
49	0.1264	0.1458	-0.2936	-0.3308	-0.0179	0.0855
50	0.1264	0.1458	-0.2936	-0.3308	-0.0179	0.0855

Table 6: The rank of items recommended for u_3 at each step.

5 Convergence on real data

In the paper, we greatly over-specified the number of steps to help ensure convergence to a stationary distribution. When the difference between the t-th updated probability to the $(t-1)$-th probability is 0, we say that the algorithm has converged. Below, we show convergence plots for some genes from a single patient. The plots show that we tend to see convergence by the 50-th iteration, confirming that the 10,000 step hyper-parameter is safely over-specified.

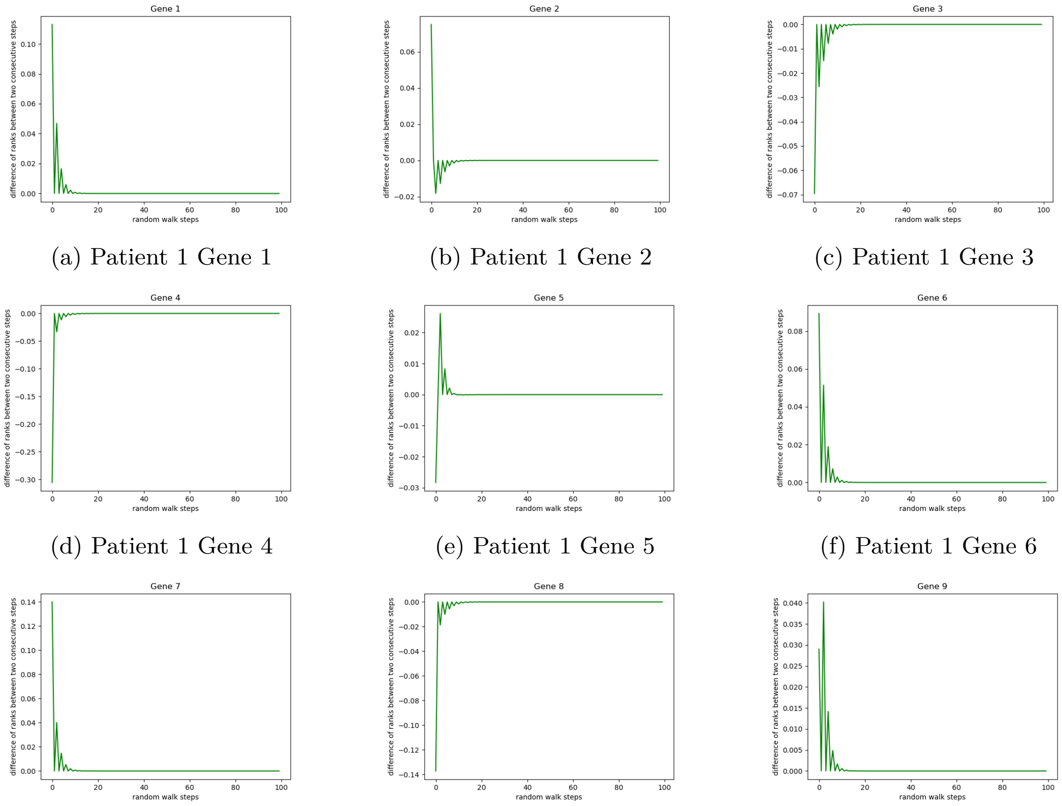

(a) Patient 1 Gene 1 (b) Patient 1 Gene 2 (c) Patient 1 Gene 3

(d) Patient 1 Gene 4 (e) Patient 1 Gene 5 (f) Patient 1 Gene 6

(g) Patient 1 Gene 7 (h) Patient 1 Gene 8 (i) Patient 1 Gene 9

Figure 2: Convergence plot of a patient for gene 1 to gene 9 genes over 100 random walk iterations.

References

[1] Yu-Chih Chen, Yu-Shi Lin, Yu-Chun Shen, and Shou-De Lin. A modified random walk framework for handling negative ratings and generating explanations. *ACM transactions on Intelligent Systems and technology (tISt)*, 4(1):12, 2013.
